# Perinatal outcomes of infants with congenital limb malformations: an observational study from a tertiary referral center in Central Europe

**DOI:** 10.1186/s12884-020-2720-x

**Published:** 2020-01-13

**Authors:** Alex Farr, Eva Wachutka, Dieter Bettelheim, Karin Windsperger, Sebastian Farr

**Affiliations:** 1 Department of Obstetrics and Gynecology, Division of Obstetrics and feto-maternal Medicine, Medical University of Vienna, Waehringer Guertel 18–20, A-1090 Vienna, Austria; 2 Department of Pediatric Orthopedics and Adult Foot and Ankle Surgery, Orthopedic Hospital Speising, Vienna, Austria

**Keywords:** Congenital, Limb malformation, Perinatal outcome, Fetal syndrome, Clubfoot

## Abstract

**Background:**

Congenital limb malformations are rare, and their perinatal outcomes are not well described. This study analyzed the perinatal outcomes of infants with congenital limb malformations.

**Methods:**

All infants with congenital limb malformations who underwent prenatal assessment and delivery at our tertiary referral center from 2004 through 2017 were retrospectively identified. Neonatal outcome parameters were assessed, and the predictors of worse perinatal outcomes were determined.

**Results:**

One hundred twenty-four cases of congenital limb malformations were identified, of which 104 (83.9%) were analyzed. The upper limb was affected in 15 patients (14.4%), the lower limb in 49 (47.1%), and both limbs in 40 (38.5%) patients. A fetal syndrome was identified in 66 patients (63.5%); clubfoot and longitudinal reduction defects were the most frequent malformations. In total, 38 patients (36.5%) underwent termination, seven (6.7%) had stillbirth, and 59 (56.7%) had live-born delivery. Rates of preterm delivery and transfer to the Neonatal Intensive Care Unit were 42.4 and 25.4%, respectively. Localization of the malformation was a determinant of perinatal outcome (*P* = .006) and preterm delivery (*P* = .046).

**Conclusions:**

Congenital limb malformations frequently occur bilaterally and are associated with poor perinatal outcomes, including high rates of stillbirth and preterm delivery. Multidisciplinary care and referral to a perinatal center are warranted.

## Introduction

Congenital malformations are rare and, to the best of our knowledge, perinatal outcomes of the affected patient population have not yet been described in detail. The European Surveillance of Congenital Anomalies (EUROCAT), a network that gathers data from approximately 1.7 million births annually in 23 countries, reported a prevalence of all types of major congenital anomalies of 23.9 per 1000 births for the years 2003 through 2007, 80% of which were live births, 2% were stillbirths, and 18% were terminated pregnancies [1]. The rate of congenital limb defects has been reported to be 3.8 per 1000 births [2, 3]. According to Bedard et al. [4], the lower limbs are less commonly affected than the upper limbs, and 10.8% of affected infants have malformations of both upper and lower limbs. However, it has to be mentioned that frequent lower limb diagnoses such as *talipes equinovarus* were not considered in this study, possibly resulting in a lower reported incidence of lower limb malformations in their study. A total of 5.4% of cases have been reported to be associated with chromosomal disorders, demonstrating associated cardiovascular, urinary tract, and digestive anomalies. Some studies have specifically examined the epidemiology of congenital upper limb malformations (ULM) [5]. Ekblom et al. [6] reported an incidence of 21.5 per 10,000 live births, with the category “failure of differentiation” being the largest subgroup.

To date, there have been very few studies that have examined the perinatal outcomes associated with congenital limb malformations. In a previously published study, the perinatal mortality of cases with ULM was reported to be 137 per 10,000 live births, compared with an overall infant mortality of 3.7 per 10,000 live births [7]. Zelop et al. [8] reported that the majority of patients examined did not survive (i.e., terminated pregnancies, neonatal deaths, and fetal demise). In the non-survivor group, 59% had aneuploidy detected by karyotype analysis. The researchers found that, when considering isolated malformations versus malformations with associations, outcomes in case of isolated malformations were pregnancy termination in 41% and survival in 58%, whereas in the group with associations, 84% were terminated pregnancies, 1% underwent fetal demise, 10% died postnatally, and 4% survived [8, 9]. Kutuk et al. [10] reported that chromosomal status was predictive of the neonatal outcome.

A glance at the currently available literature reveals that researchers have been focusing on the perinatal outcomes associated with clubfeet [11–14], followed by toe/finger malformations [15], amniotic band syndrome [16], and other various pathologies, including limb reduction defects, arthrogryposis, polydactyly, and abnormal hand position [17]. Some reports have focused on the prenatal diagnostic assessment rather than the obstetric outcomes in this selected patient cohort [18, 19]. Because of the high number of terminated pregnancies and lack of postnatal records, the available data are however limited. Therefore, in the present study, we aimed to contribute to the sparse available body of literature, offering data on congenital limb malformations at a large tertiary referral center in Austria. The knowledge gained from this study could help to improve multidisciplinary care and assist parents in the decision-making process.

## Materials and methods

### Setting and procedure

We conducted a retrospective observational study to identify cases with a prenatal diagnosis of congenital limb differences. The institutional database was searched during the period from January 1, 2004 to December 31, 2017. The Department of Obstetrics and Gynecology of the Medical University of Vienna is an internationally recognized tertiary center with a highly specialized maternal-fetal care unit. We included all pregnant women with no limitations to age and/or ethnicity; fetuses needed to be diagnosed with at least one limb malformation during the prenatal sonogram at our tertiary referral center. The only exclusion criterion was unclear imaging results. Of note, prenatal diagnosis is optional and not covered by insurance in Austria; termination of pregnancy (TOP) is allowed during the first three months after conception, and after that there needs to be a serious reason for TOP that is adjudicated by a multidisciplinary board.

### Data acquisition

Data of infants with congenital limb malformations who underwent prenatal assessment or delivery at our institution were analyzed. First, a manual review of eligible cases was performed using the perinatal database PIA Viewpoint (GE Healthcare) and AKIM software (SAP). Cases were reviewed for accuracy. The perinatal data collected included the following parameters: other case of pregnancy with fetal limb malformation in the mother’s medical record, maternal age at diagnosis, gestational age at diagnosis, date of last menstruation, estimated date of delivery, highest level of maternal education, nicotine use, maternal height, maternal weight, conception and artificial reproductive treatment, previous preterm delivery, gravidity, parity, details on the malformation (extremity, side, localization), sonographic findings, fetal syndrome, nuchal translucency (NT) at first trimester screening, fetal magnetic resonance imaging (fMRI), noninvasive prenatal testing (NIPT), amniocentesis (AC), chorionic villus sampling (CVS), cordocentesis, chromosomal testing, multiple pregnancy, termination of pregnancy (TOP), induced fetal demise (feticide), intrauterine fetal demise (IUFD), mode of delivery, date of birth, gestational age at delivery/abortion, live birth or stillbirth, preterm delivery, neonatal gender, neonatal birthweight, percentile of birthweight, neonatal length, percentile of length, neonatal head circumference, percentile of head circumference, Apgar scores (at 1, 5, and 10 min), umbilical cord arterial pH value, umbilical cord base excess value, transfer to the neonatal intensive care unit (NICU), and loss to follow-up. In some cases, it was necessary to request additional information from other hospitals.

### Outcome parameters

For statistical analyses, we divided the cases into three groups: upper limb malformations (ULM), lower limb malformations (LLM), and both upper and lower limb malformations (BLM). The pregnancy outcome (TOP versus IUFD versus live birth) served as the primary outcome parameter. Secondary outcome parameters included the rate of preterm delivery, Apgar score, birthweight, umbilical cord arterial pH value, gestational age at delivery/abortion, and mode of delivery.

### Statistical analysis

Parametric data are presented as means with standard deviations as well as minimum and maximum values. The Welch *t* test was used to compare continuous data, while the Fisher exact test was used to compare categorical data. Correlations between variables were determined using the Spearman correlation test. Data were analyzed using SPSS version 23.0 (IBM) with the level of statistical significance set to .05.

## Results

A total of 124 cases with congenital limb malformations were identified, of which 104 (83.9%) were evaluated and 20 (16.1%) were lost to follow-up. The mean maternal age at the time of diagnosis was 30.2 ± 6.5 years, with a mean gestational age of 20.5 ± 5.4 weeks. Of the 104 cases, seven (6.7%) were twin pregnancies. Maternal characteristics are provided in Table 1.

**Table 1:** Maternal characteristics of 104 followed cases with congenital fetal limb malformations

	Mean ± SD N (%)
Maternal age at diagnosis (years)	30.2 ± 6.5
Gestational age at diagnosis (weeks)	20.5 ± 5.4
Educational level	
primary/high school	95 (91.3)
higher education	9 (8.7)
Consanguinity of parents	
consanguinity	7 (6.7)
no consanguinity	97 (93.3)
Smoking at the beginning of pregnancy	
smoking	17 (16.3)
no smoking	87 (83.7)
Artificial reproductive treatment	
hormonal stimulation	2 (1.9)
IVF/ICSI	7 (6.7)
no artificial reproductive treatment	95 (91.4)
Previous preterm delivery	
history of preterm delivery	13 (12.5)
no history of preterm delivery	74 (71.2)
not available	17 (16.3)

*IVF* in-vitro fertilization; *ICSI* intracytoplasmic sperm injection

Limb malformations affected the right side in 11 patients (10.6%), the left side in 13 (12.5%), and both sides in 80 (76.9%). The upper limb was affected in 15 patients (14.4%), the lower limb in 49 (47.1%), and both limbs in 40 (38.5%). Clubfoot was the most frequently isolated malformation with 56 affected infants (53.8%), followed by 12 (11.5%) with longitudinal reduction defects of the upper limb, and 12 (11.5%) with longitudinal reduction defects of the lower limb. The residual cases included ectrodactyly (3/104; 2.9%), syndactyly (1/104; 1%), amelia/phocomelia (4/104; 3.8%), arthrogryposis (6/104; 5.8%), dysmelia (1/104; 1%), finger abnormalities (3/104; 2.9%), peromelia (3/104, 2.9%), polydactyly (1/104; 1%), and other malformations (2/104; 1.9%).

After sonographic diagnosis of a congenital, fMRI was performed in 58 cases (55.8%), AC in 28 (26.9%), CVS in 31 (29.8%), and NIPT in four (3.8%); in one case, both CVS and AC were performed. A total of 63 cases (60.6%) underwent first trimester screening with NT scan, with a mean NT of 2.6 ± 1.8 mm. Thirty-one infants (29.8%) were female, 52 (50%) were male, and in 21 cases (20.2%), gender was either not registered or genitalia were indifferent.

**Fig. 1:**
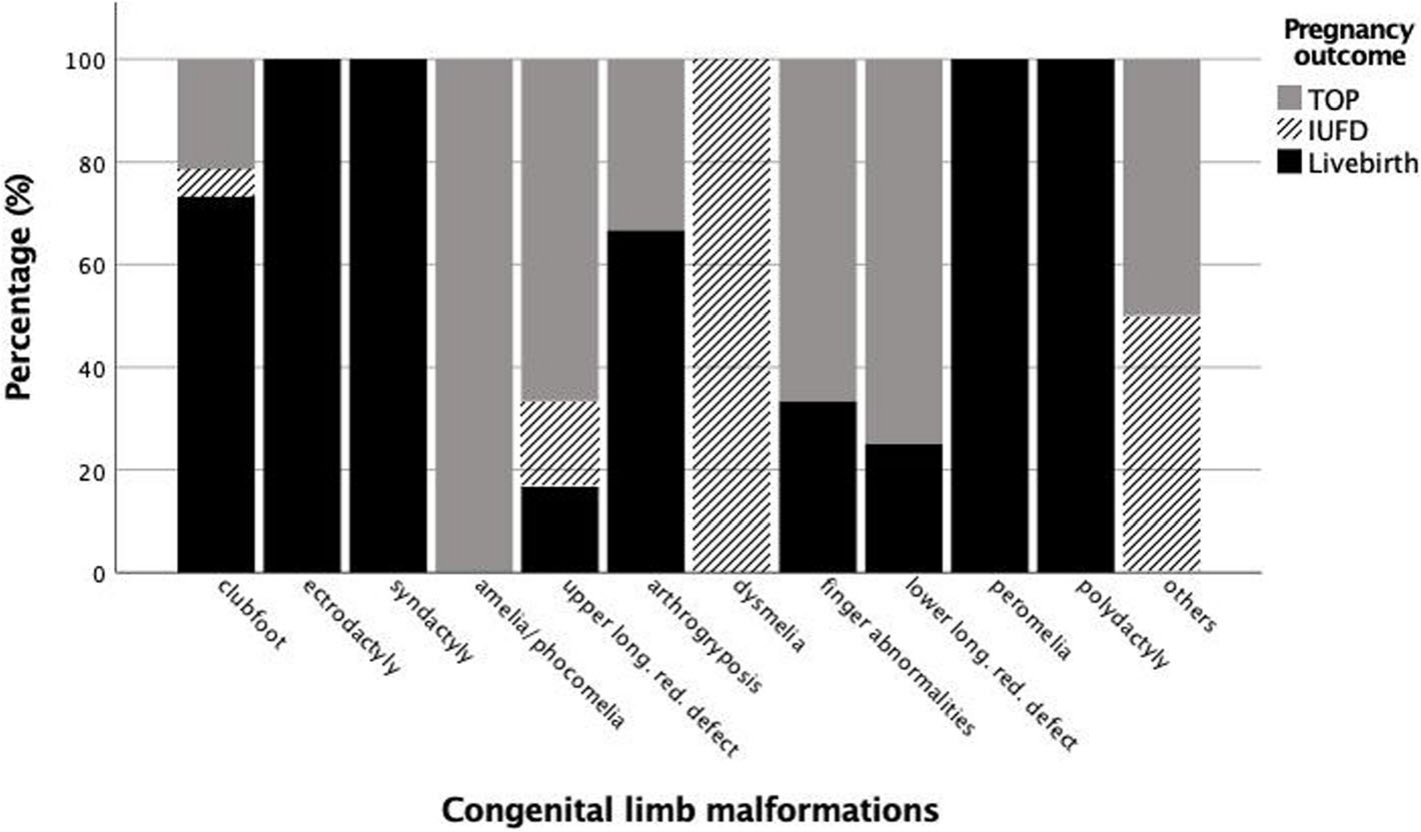
Outcomes according to the diagnosis of the congenital limb malformation

Of the 104 followed cases, 38 (36.5%) underwent TOP, of which ten (26.3%) were terminated by induced fetal demise (feticide); 59 (56.7%) were born live, including two live-born cases after termination attempts using mifepristone followed by misoprostol. In detail, 34 cases (57.6%) involved term and 25 (42.4%) involved preterm delivery. Of the live-born infants, 28 (47.5%) were born vaginally, two (3.4%) by instrumental delivery, and 29 (49.1%) by caesarean section (Table 2). A fetal syndrome was identified in 66 cases (63.5%). Outcomes according to the diagnosis of the limb malformation are displayed in Fig. 1.

**Table 2:** Perinatal outcomes of 59 live-born infants with congenital limb malformations

	Mean ± SDMedian (Min–Max) N (%)
Gestational age at delivery (weeks)	36.9 ± 4.8
Birthweight at delivery (grams)	2566 ± 943
Umbilical cord arterial pH	7.27 ± 0.07
Umbilical cord base excess	−2.6 ± 3.8
Preterm delivery rate	
term delivery	34 (57.6)
preterm delivery^†^	25 (42.4)
Mode of delivery	
spontaneous vaginal	28 (47.5)
instrumental	2 (3.4)
cesarean section	29 (49.1)
Apgar score	
at 1 min	9 (1–9)
at 5 min	10 (0–10)
at 10 min	10 (0–10)
NICU transfer	
transfer to NICU	15 (25.4)
no transfer to NICU	39 (66.1)
comfort terminal care	5 (8.5)

*NICU* neonatal intensive care unit; ^†^including live-born cases with iatrogenic preterm delivery

When comparing the outcomes of ULM, LLM, and BLM, we found a TOP rate of 33.3% in ULM, 20.4% in LLM, and 57.5% in BLM. IUFD occurred in 13.3% in ULM, 6.1% in LLM, and 5% in BLM. Live birth rate was 53.4% in ULM, 73.5% in LLM, and 37.5% in BLM. The median (Min-Max) Apgar scores were significantly different in the 104 followed cases with available perinatal outcomes (*P =* .002), as displayed on Table 3.

**Table 3:** Perinatal outcomes of 104 followed cases according to localization of the limb malformation

	Upper limb (ULM, *N* = 15)	Lower limb (LLM, *N* = 49)	Upper/lower limb (BLM, *N* = 40)	*P*
	N (%)Median (Min-Max)			
Pregnancy outcome				
TOP	5/15 (33.3)	10/49 (20.4)	23/40 (57.5)	.006
IUFD	2/15 (13.3)	3/49 (6.1)	2/40 (5)	
Live birth	8/15 (53.4)	36/49 (73.5)	15/40 (37.5)	
Outcomes of live-born				
term delivery	2/8 (25)	25/36 (69.4)	7/15 (46.7)	.046
preterm delivery	6/8 (75)	11/36 (30.6)	8/15 (53.3)	
Apgar score				
at 1 min	2 (0–9)	8 (0–9)	0 (0–9)	.002
at 5 min	2 (0–10)	10 (0–10)	0 (0–10)	
at 10 min	3 (0–10)	10 (0–10)	0 (0–10)	
NICU transfer				
transfer to NICU	4/8 (50)	8/36 (22.2)	3/15 (20)	n.s.
no transfer to NICU	4/8 (50)	27/36 (75)	8/15 (53.3)	
comfort terminal care	0/8 (0)	1/36 (2.8)	4/15 (26.7)	

*TOP* termination of pregnancy; *IUFD* intrauterine fetal demise; *NICU* neonatal intensive care unit; *n.s.* not statistically significant

When applying the Chi-squared test, we found a statistically significant association between the pregnancy outcome (TOP versus IUFD versus live birth) and presence of a fetal syndrome (*P* < .001). Localization of the limb malformation (upper versus lower versus both) was a significant determinant of the pregnancy outcome (*P* = .006; Table 3). No statistically significant association was found between the pregnancy outcome and artificial reproductive treatment (*P* = .153), side of the limb malformation (*P* = .241), gender (*P* = .192), or nicotine use (*P* = .11). Infants with a syndrome had a significantly lower birth weight (1328 ± 1174 g with syndrome versus 2252 ± 1271 g without syndrome; *P* = .001). Perinatal outcomes according to the localization of the limb malformation are shown in Table 3.

## Discussion

Congenital limb malformations are rare, and the clinical presentations range from isolated malformations to complex syndromes and aneuploidies. Since there are limited data available, the aim of this study was to analyze the perinatal outcomes of infants with congenital limb malformations.

According to our data, cases with congenital limb malformations were shown to have a rather high rate of induced abortion, which is understandable, given the selected patient group and our high level of perinatal care. This assumption is supported by our finding that only 83.9% of cases had available follow-up data. Apart from this theory, there remains the possibility that patients were treated at outpatient departments or were only seeking a second opinion at our department, making it impossible to follow their cases. Our study showed a higher loss-to-follow-up rate than those of studies by Zelop et al. [8] and Sharma et al. [14], who reported 96.3 and 88.5% follow-up rates, respectively. A possible explanation could be that the followed cases had lower rates of syndromes and bilateral malformations as well as a higher rate of both upper and lower limb malformations. In these cases, parents were more likely to terminate the pregnancy.

For statistical analysis, we stratified perinatal outcomes by IUFD, TOP, or live birth, and found that younger maternal age was associated with more favorable outcomes; this might be related to the higher rate of more complex syndromes in older women (data not shown) [11]. According to the literature, the mean maternal age of women with a congenital malformation of the fetal limb ranged between 22.1 and 31.5 years [9, 10, 13, 20]. Of note, positive family history of congenital limb malformations showed no association with the pregnancy outcome (i.e., IUFD versus TOP versus live birth). When comparing our results with those reported in the literature, we found a higher rate of family history compared with those of studies by Paladini et al. [9] and Kutuk et al. [10], who reported rates of 8 and 9.8%, respectively. It can be argued that our study cohort included a high number of cases with clubfeet compared with these studies that focused on the outcomes associated with ULM. Sharma et al. [14] reported a family history rate of 16.3%, while Sharon-Weiner et al. [11] reported rates of 9.2 and 4.6% for parental and sibling affectedness, respectively. These studies investigated the outcomes of fetuses with clubfeet and found a higher family history rate, supporting this theory.

We found that bilateral limb malformations were associated with worse outcomes and higher termination rates compared with unilateral cases; the TOP rate was significantly higher, and the live birth rate was significantly lower in BLM compared to ULM and LLM. In our study, the IUFD rate was significantly higher in the ULM group compared to the others. The live birth rate was the highest in isolated LLM, followed by ULM and BLM, which is consistent with the findings of Bakalis et al. [12] and Sharma et al. [14]; this might most likely be attributed to the high incidence of isolated clubfeet in the LLM group.

The most commonly occurring malformation was clubfoot in all three groups (IUFD, TOP and live birth), a finding that accords with the existing literature reporting that clubfeet are the most common pediatric foot malformations with a prevalence of 0.6 to 1.5 per 1000 live births [21]. The second and third most common malformations in our study were longitudinal reduction defects of the upper limb and of the lower limb, respectively, in all groups. Our findings lead to the assumption that clubfoot, in particular, is associated with good obstetric outcomes, as we found a high proportion of clubfeet in live birth cases.

We also found higher rates of IUFD and TOP in cases with a fetal syndrome, as it has previously been described [7–12, 14, 16, 17]. A majority of existing studies [8, 11, 13, 14, 17, 18] reported trisomy 18 as the most common aneuploidy associated with congenital limb malformations, and this was consistent with our data. Paladini et al. [9] and Dicke et al. [17] reported trisomy 13 to be the second most common aneuploidy associated with limb malformations. With regard to the perinatal outcome data of live-born infants, the live birth cases in our study showed a high preterm delivery rate of 42.4%, which is remarkbly higher than the overall preterm delivery rate in Austria.

In part, the increased rate of preterm delivery in our study might be explained by contributing risk factors for preterm delivery (e.g., previous preterm delivery) that are criteria for registration for a planned delivery at our tertiary referral center [22]. Compared with the results of Sharma et al. [14], we also found a rather high preterm delivery rate in cases with isolated clubfeet, attributed to the fact that we are the largest perinatal center in the region and that cases with other comorbidities and reasons for preterm delivery were included. We are aware that many of the reported preterm deliveries might have been iatrogenic due to preeclampsia, cervical insufficiency, intrauterine growth retardation, maternal comorbidities, imminent fetal asphyxia or for various other reasons.

With regard to localization of the limb malformation, we found that the perinatal outcome was worse in cases that affected the upper or both limbs compared with isolated LLM (e.g., clubfeet) that more frequently resulted in live birth. Koskimies et al. [16] found a lower perinatal mortality rate of 13.3% in LLM compared with 14.8% in ULM. In the subgroup analysis of live birth cases, preterm delivery was found in 75% of ULM, 30.6% of LLM, and 53.3% of BLM cases, again demonstrating the superior outcomes of infants with LLM. From a clinical perspective, preterm delivery should be anticipated, including timely information of parents in cases with ULM and BLM. Evaluating the Apgar score at 5 min, a well-known short-term outcome parameter for both obstetricians and neonatologists, we found also found superior outcomes in LLM cases compared to ULM and BLM (Table 3), suggesting that isolated LLM is unlikely to worsen the neonatal outcome. Considering together the cases with NICU transfer and comfort terminal care, rates were again higher in the BLM and ULM groups than in the LLM group.

We are aware that our study has several limitations. First among them is the retrospective study design that could have led to selection bias and false conclusions. Furthermore, our observational report of cases could have benefited from a matched control group. The malformations that we reported were diagnosed using ultrasonography; fMRI reports were only occasionally available; postnatal confirmation of prenatally diagnosed malformations would have been beneficial as well as standardized fMRI measurements in all analyzed cases. Finally, we could provide neither long-term outcomes nor chromosomal testing results of all observed infants, possibly resulting in the underestimation of the number of associated syndromes, especially in the TOP and IUFD groups.

Despite these limitations, our study has strengths, including its implementation at a single tertiary center with a high number and various types of malformations. Treatment at our center was associated with examinations that were exclusively conducted by well-trained and certified examiners, which is of paramount importance in this context. Therefore, our data are homogeneous and reliable, which are particularly important in observational studies with relatively small sample sizes.

## Conclusions

We found that congenital limb malformations were associated with poor perinatal outcomes, including high rates of preterm delivery and stillbirth. Moreover, localization of the affected limb and presence of a more complex fetal syndrome were both determinants of the pregnancy outcome. The perinatal outcomes might be more favorable in non-syndromal cases and in those with isolated LLM, such as clubfoot. After prenatal diagnosis of a fetal limb malformation, further evaluation should include detection of other malformations or chromosomal disorders, as they are associated with worse outcomes. Indeed, multidisciplinary care and referral to a perinatal center are highly warranted in these cases.

## Data Availability

The datasets used and/or analyzed during the current study are available from the corresponding author on reasonable request.

## Abbreviations

AC: Amniocentesis
BLM: Upper and lower limb malformation
CVS: Chorionic villus sampling
EUROCAT: European Surveillance of Congenital Anomalies
fMRI: Fetal magnetic resonance imaging
IUFD: Intrauterine fetal demise
LLM: Lower limb malformation
NICU: Neonatal Intensive Care Unit
NIPT: Noninvasive prenatal testing
NT: Nuchal translucency
TOP: Termination of pregnancy
ULM: Upper limb malformation
